# Disseminated Nocardia cyriacigeorgica Infection Disguised as a Metastatic Adrenal Gland Malignancy in a Healthy Patient

**DOI:** 10.7759/cureus.63693

**Published:** 2024-07-02

**Authors:** Mathew Daniel, Sadik Salman, Benjamin Adam

**Affiliations:** 1 Division of General Internal Medicine, Department of Medicine, University of Alberta, Edmonton, CAN; 2 Department of Laboratory Medicine and Pathology, University of Alberta, Edmonton, CAN

**Keywords:** ring-enhancing brain lesion, disseminated nocardia, immunocompetent, adrenal gland abscess, nocardia cyriacigeorgica

## Abstract

*Nocardia*, typically recognized as an uncommon opportunistic pathogen affecting immunocompromised individuals, has also been documented in various case reports involving infections in immunocompetent hosts. Transmission occurs through inhalation or inoculation into compromised skin. Subsequently, it can lead to disseminated infection via hematogenous spread, affecting nearly any organ with a particular affinity for the central nervous system. Dissemination to the adrenal glands is extremely rare, with only a few cases reported. In this report, we present a rare case of disseminated *Nocardia cyriacigeorgica*, initially resembling a metastatic adrenal gland malignancy in an otherwise healthy individual. The patient presented with non-specific symptoms, had multiple sets of negative blood cultures, clinical findings suggestive of an underlying adrenal gland malignancy, and lacked identifiable risk factors for *Nocardia*, creating a significant diagnostic challenge. Additionally, we review the existing literature on nocardiosis involving the adrenal glands. This case marks the third reported instance of a *Nocardia cyriacigeorgica* adrenal gland abscess in the literature.

## Introduction

*Nocardia*, although typically thought of as an opportunistic infection impacting the immunocompromised, is well documented in the literature as affecting immunocompetent individuals as well [1-7]. This Gram-positive, branching, filamentous bacterium has mildly acid-fast staining and is commonly found in soil, decaying vegetation, aquatic environments, sand, dust, wastewater, swimming pools, and manure [5]. Transmission typically occurs through inhalation or inoculation into compromised skin [2,5]. Once localized, it has the potential to disseminate via the bloodstream to various organs, with a particular affinity for the central nervous system (CNS) [2]. Dissemination to the adrenal glands is exceptionally rare, with only a few cases reported in the literature [1-4,6-12].

We report a rare case of disseminated nocardiosis that initially presented as a metastatic adrenal gland malignancy in a healthy patient with no apparent risk factors. The clinical presentation suggested malignancy rather than infection, highlighting the diagnostic challenges that can be associated with *Nocardia*. This case underscores the importance of maintaining a high index of suspicion for *Nocardia* infections. Additionally, we review the current literature on nocardial adrenal gland abscesses, noting that this is the third documented case of a *Nocardia cyriacigeorgica* adrenal gland abscess.

## Case presentation

A healthy 72-year-old woman, previously admitted to the general internal medicine (GIM) department for left upper quadrant abdominal pain and evaluation of a newly discovered 6 cm left adrenal mass, returned to the emergency department 10 days after discharge, showing signs of failure to thrive and confusion. She was accompanied by her sister, who reported that the patient had appeared normal a few days earlier but had since become progressively lethargic and confused with reduced oral intake.

The patient had a lifelong history of abstaining from smoking and alcohol consumption. She was a retired stay-at-home mom currently living alone, with no past medical history or prior hospitalizations, and resided within the city in a middle-class neighborhood. Her medications included estradiol and a recent prescription for tramadol-acetaminophen from her previous hospitalization.

**Figure 1:**
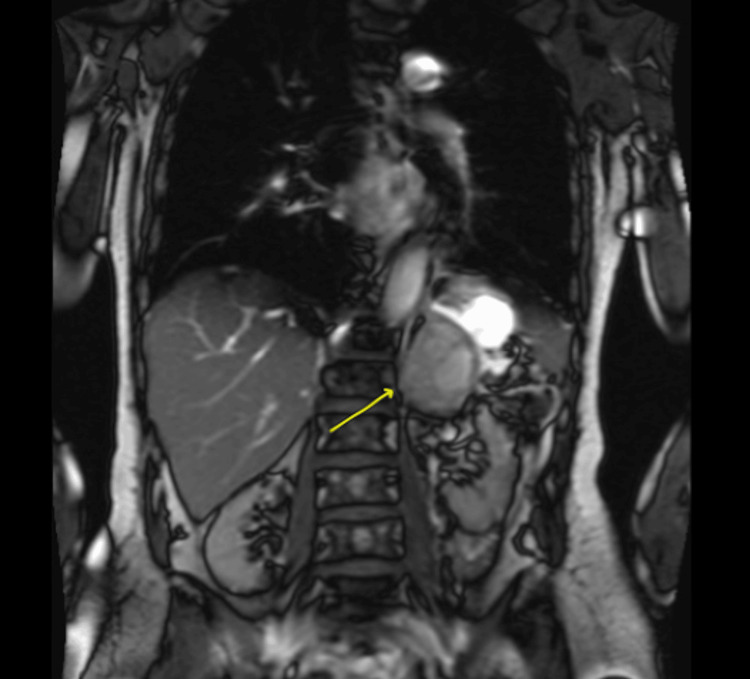
A coronal image of the unenhanced MR abdomen displays a 5.6 cm by 4.5 cm by 5.8 cm (ML by AP by CC) left adrenal mass, accompanied by minimal surrounding stranding AP: anterioposterior, ML: mediolateral, CC: craniocaudal

Previous investigations from her first admission revealed the adrenal mass to be non-functional, with normal 24-hour urine metanephrines, aldosterone to renin ratio, adrenocorticotropic hormone levels, and mildly elevated morning cortisol, likely due to acute illness at the time. During this admission, she was noted to have leukocytosis (WBC 14.5x10^9^ cells/L, normal 4-11x10^9^ cells/L) and elevated CRP (251 mg/L, normal <8.1 mg/L), but was afebrile and had negative blood and urine cultures and sexually transmitted blood-borne infections panel. An MRI of her abdomen showed an approximately 6 cm left adrenal mass-like hemorrhage with stranding (Figure 1). There was no history of trauma or anticoagulant use. She was discharged with instructions to follow up with general surgery and to have a repeat CT of the abdomen in three weeks.

Upon this presentation to the emergency department, she was afebrile and hemodynamically stable, appearing comfortable. Although she was not fully alert, she responded to minor stimulation and answered basic questions. She denied any recreational drug use, misuse of her newly prescribed pain medication, or any symptoms suggesting an infection. She had ongoing left upper quadrant abdominal pain with no acute changes. There was no recent travel, unusual exposures, use of over-the-counter or herbal medications, or contact with sick individuals.

On the physical exam, she had no nuchal rigidity or jolt accentuation. Her pupils were equal and reactive to light. She exhibited weakness but had no focal motor deficits, with intact sensation and normal reflexes. Heart sounds were normal without murmurs, and clinically, she appeared hypovolemic, with a flat jugular venous pressure and dry mucous membranes. Her lungs were clear on auscultation, and her abdomen was soft, non-tender, non-distended, and without palpable organomegaly. Her extremities were warm, and she had no signs suggestive of cellulitis.

**Figure 2:**
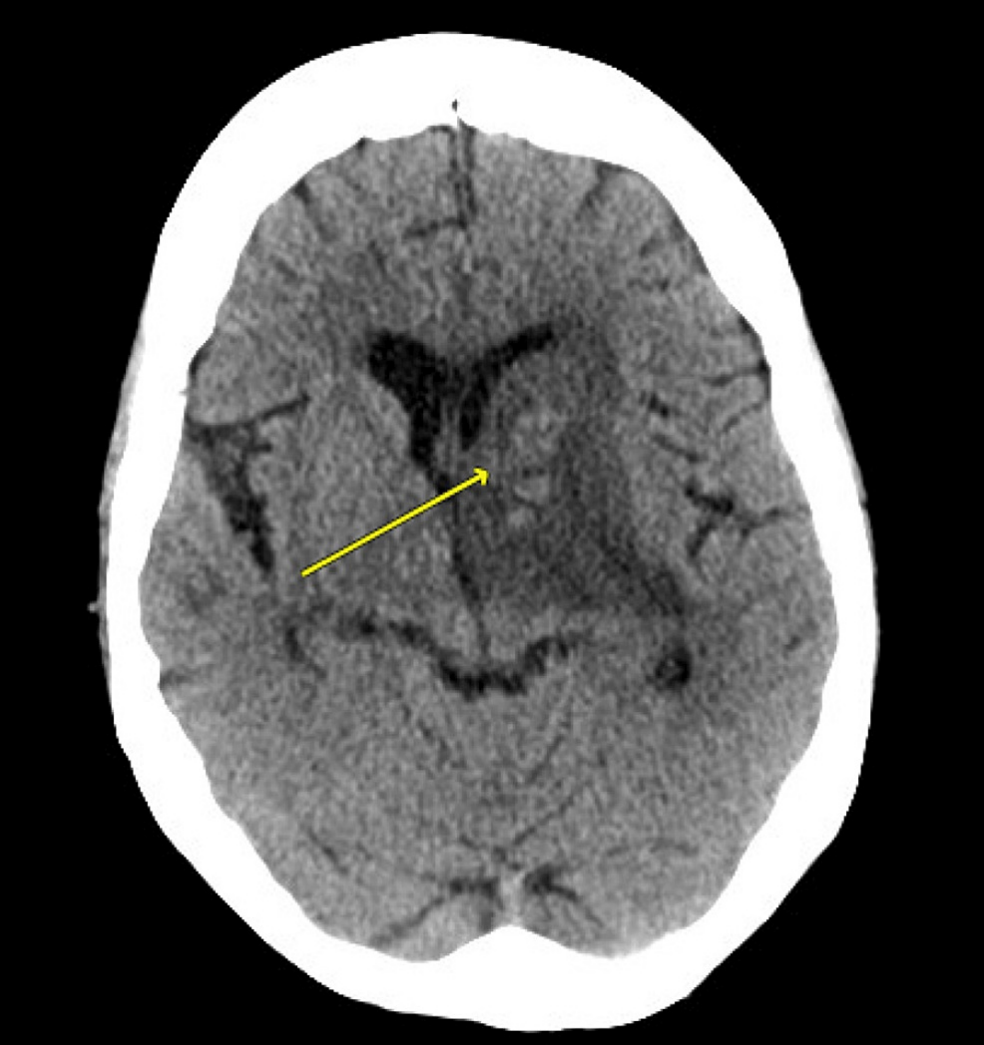
An axial image from the unenhanced CT scan of the head reveals significant central cerebral edema affecting the left basal ganglia and thalamus CT: computed tomography

A CT/CTA of her head and neck suggested a large deep left middle cerebral artery (MCA) non-hemorrhagic infarct with significant central cerebral edema involving the left basal ganglia (Figure 2). However, the acute stroke team did not believe her symptoms indicated an acute stroke due to the lack of lateralizing symptoms and atypical radiographic findings for MCA strokes. They were instead concerned about metastases due to her new left adrenal gland mass. Other pertinent laboratory investigations revealed that compared to her previous admission, her leukocytosis and CRP had improved but remained elevated (WBC count 14.5x10^9^ cells/L -> 13.3x10^9^ cells/L, CRP 251 mg/L -> 231 mg/L), and she had a decrease in her hemoglobin by 13 points (104 g/L -> 91 g/L, normal 120-160 g/L). She had no other significant metabolic derangements, with normal renal function, electrolytes, extended electrolytes, liver enzymes, liver function tests, TSH, and ammonia levels.

A repeat CT abdomen, prompted by the unexplained 13-point hemoglobin drop, showed a marked increase in the size of the left adrenal mass-like hemorrhage, now approximately 11 cm with lobular, multi-septated enhancing components, and a new left renal vein thrombus. She was readmitted to GIM, and blood and urine cultures were collected. She was started on intravenous dexamethasone 8 mg twice daily given the significant cerebral edema, with consultations requested from general surgery and neurosurgery. Empiric anticoagulation was deferred due to the potential for an adrenal biopsy.

**Figure 3:**
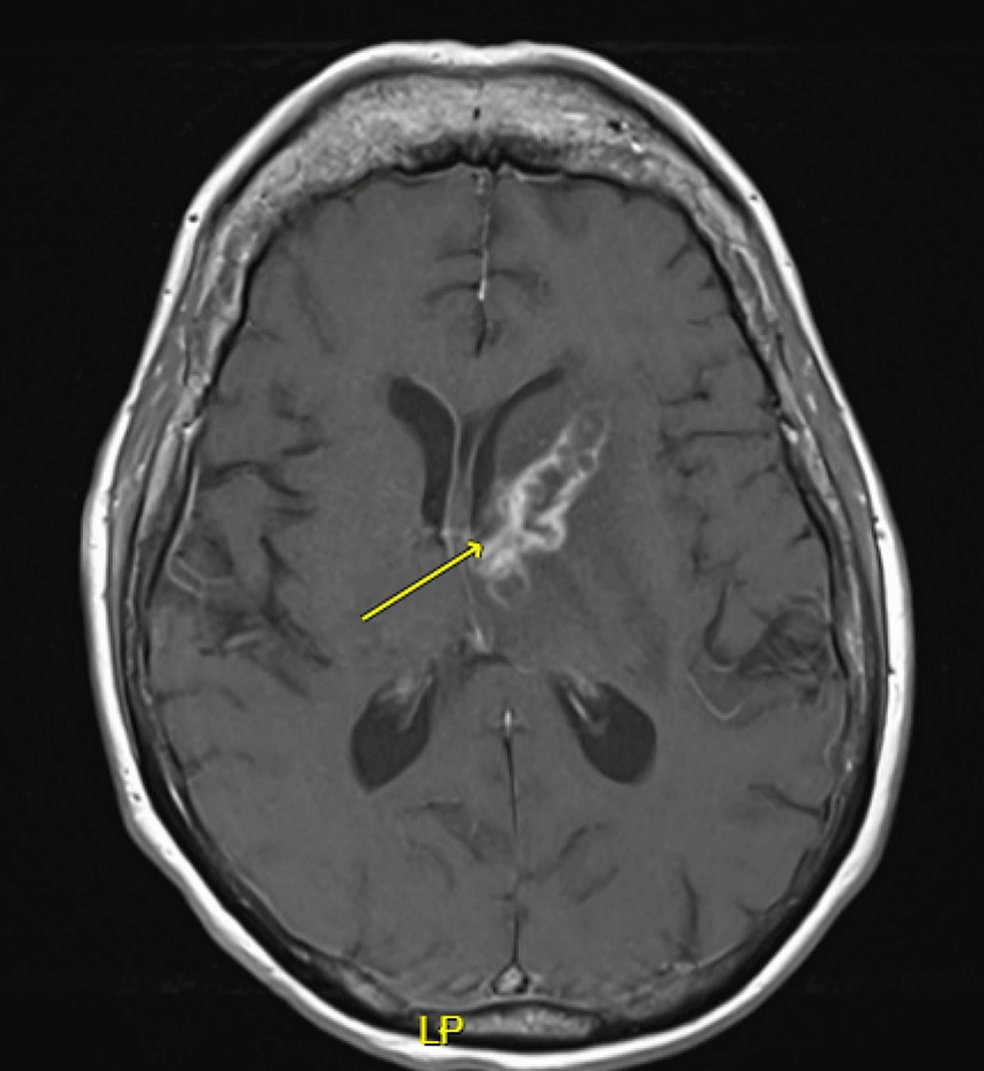
An axial T1-weighted MRI brain image displays a lesion with peripheral enhancement located in the left basal ganglia. Additionally, a smaller, similar lesion in the right temporal lobe is not visible in this image. Mild to moderate vasogenic edema was observed surrounding both lesions on T2-weighted imaging MRI: magnetic resonance imaging

The next day, an enhanced MRI of the brain revealed two rim-enhancing lesions with central diffusion restriction in the left basal ganglia and right temporal lobe, accompanied by moderate vasogenic edema (Figure 3). A staging CT chest was performed due to the suspected metastatic adrenal malignancy and returned normal.

At this stage, a unifying diagnosis was unclear. There was no tissue diagnosis confirming malignancy, and while leukocytosis and CRP, along with central diffusion restriction, suggested an infectious etiology, multiple sets of negative blood cultures, improving leukocytosis without antibiotics, and a lack of fever argued against this. Moreover, the adrenal gland imaging findings (solid components within the adrenal mass, associated hemorrhage, renal vein thrombus) suggested malignancy as the most likely diagnosis.

Neurosurgery advised that no neurosurgical intervention was warranted and recommended repeating blood cultures and continuing the steroids for her vasogenic edema. General surgery proposed a fine needle aspiration biopsy of the adrenal mass, but the patient's condition deteriorated over the following days with seizures and a worsening level of consciousness, leading to palliative care given the presumed metastatic malignancy and her overall clinical picture. She passed away peacefully shortly thereafter.

**Figure 4:**
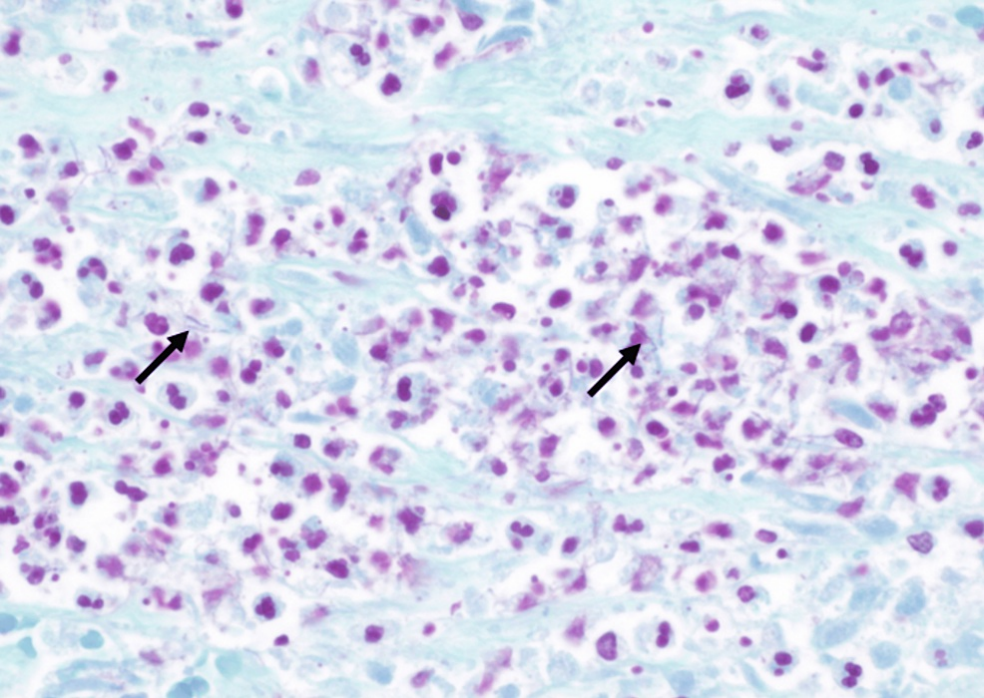
Gram stain from the adrenal abscess revealing Gram-positive, branching, filamentous bacteria consistent with Nocardia

**Figure 5:**
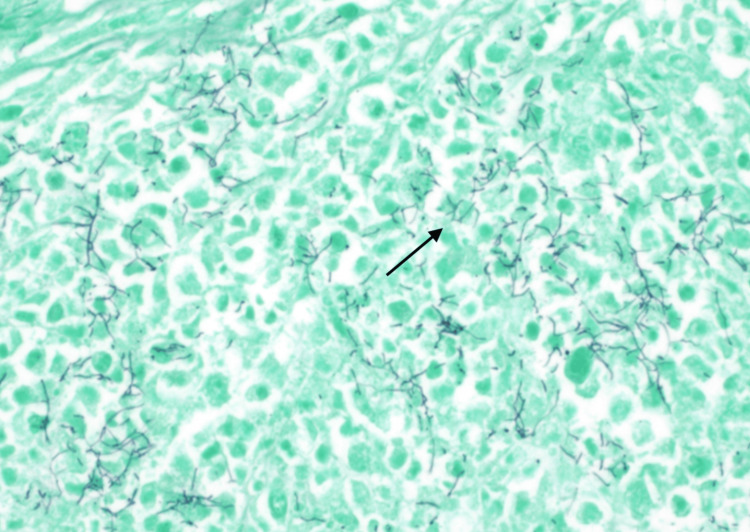
A GMS stain of the adrenal gland abscess reveals thin, dark, branching filamentous structures consistent with Nocardia GMS: Gomori methenamine silver

An autopsy requested by her family revealed that the adrenal mass and brain lesions were abscesses containing *Nocardia*, confirmed through staining (Figures 4-5). Additionally, there were numerous small abscesses in the lungs, a pancreatic abscess, and an omental abscess, all containing *Nocardia*. A pre-mortem blood culture drawn four days before her death returned positive for *Nocardia cyriacigeorgica* shortly after she passed away, with a time to positivity of three days, 11 hours, and 20 minutes.

## Discussion

*Nocardia* infections are often considered opportunistic, primarily affecting immunocompromised individuals, notably those with compromised cell-mediated immunity like HIV patients, solid organ or hematopoietic stem cell transplant recipients, or individuals on long-term high-dose corticosteroids [13,14]. However, as many as one-third of patients affected are immunocompetent [13,14]. Transmission of *Nocardia* typically occurs through inhalation or skin inoculation via penetrating trauma or contamination from soil, water, or dust [2]. The lungs are the most common site of infection, followed by cutaneous infections [15].

While *Nocardia *usually causes localized infections at the point of entry, disseminated infection, defined as an infection in two or more non-contiguous sites, occurs through hematogenous spread [5]. The CNS is the most frequent site of dissemination, reported in 20-58% of nocardial infections [5]. In patients with CNS nocardiosis, MRI often reveals ring-enhancing lesions with surrounding vasogenic edema. However, the diagnostic spectrum for peripherally enhancing lesions is broad and encompasses various infectious (e.g., bacterial, fungal, parasitic) and non-infectious causes (e.g., neoplasm) [5].

Our case presents a diagnostic challenge involving an immunocompetent patient with disseminated *Nocardia cyriacigeorgica* affecting multiple organs (lungs, blood, adrenal gland, pancreas, omentum, and brain) which initially presented as a suspected metastatic adrenal gland malignancy. A recent retrospective review of *Nocardia *isolates in China from 2009 to 2021 revealed *Nocardia cyriacigeorgica* is the second most commonly implicated species (28.2%) [16]. Presumably, the patient in our case contracted *Nocardia* through inhalation, given the numerous small lung abscesses, followed by hematogenous spread to the adrenal gland and brain, which manifested radiographically as a left adrenal mass and ring-enhancing brain lesions with vasogenic edema. The other intra-abdominal and intrathoracic findings were not found on imaging that was done prior to the autopsy.

Our initial suspicion leaned toward an underlying adrenal malignancy rather than a disseminated infection. This suspicion stemmed from several factors: the patient's lack of fever, stable vital signs, improving white blood cell count and inflammatory markers without antibiotic therapy, repeated negative blood cultures (except for the one blood culture that returned positive shortly after she had passed), the radiographic appearance of the adrenal mass (solid components with hemorrhagic involvement), and the presence of a renal vein thrombus, presumed to be due to underlying malignancy-related hypercoagulability.

The occurrence of nocardiosis involvingthe adrenal glands is extremely rare. A literature review found only 11 cases of nocardial adrenal gland abscesses in PubMed (Table 1). *Nocardia farcinica* was the most frequently identified species, accounting for six out of the 11 cases. *Nocardia cyriacigeorgica* was responsible for two cases, both in immunocompetent individuals with risk factors for *Nocardia *(i.e., farmer, construction worker). Among the 11 cases, three resulted in fatalities, all of whom were immunocompromised. Disseminated disease was observed in 10 cases, with nine cases presenting with fever and two cases without fever. The cases without fever exhibited other signs of infection (i.e., respiratory distress, chest imaging infiltrates, or hemoptysis) and/or had identifiable risk factors for *Nocardia* (i.e., construction work with dust exposure or HIV). Notably, the majority of these cases detected nocardiosis* *using multiple modalities: blood cultures (7 out of 11 cases), fluid or tissue cultures (9 out of 11 cases), and/or molecular techniques (6 out of 11 cases). This is particularly important for the detection of *Nocardia*, as it is not uncommon for the disease to be present despite negative blood cultures, as evidenced in our case.

**Table 1:** Reported cases in the literature of nocardial adrenal gland abscesses mNGS: metagenomics next-generation sequencing, GMS: Gomori methenamine silver, TMP-SMX: trimethoprim-sulfamethoxazole, MALDI-TOF MS: matrix-assisted laser desorption-ionization-time of flight mass spectrometry, IVDU: intravenous drug user, ESRD: end-stage renal disease, TPN: total parental nutrition, BAL: bronchoalveolar lavage

Author	Sites of infection	Patient presentation	Patient factors	Nocardia spp.	Detection	Treatment	Outcome
Wang et al. 2024 [8]	Bacteremia, right adrenal gland abscess, left shoulder furuncle	Fever, right-sided abdominal pain	80-year-old female, disseminated non-tuberculosis mycobacteriosis, cutaneous T cell lymphoma, hypertension	Farcinica	plasma mNGS, blood culture	Imipenem, cilastatin sodium, linezolid	Deceased after less than a month of antimicrobials
Kesim et al. 2023 [9]	Lungs, right adrenal gland abscess	Hemoptysis	49-year-old male, HIV, remote tuberculosis	*Nocardia spp.* (not speciated)	Wedge resection of FDG avid lung nodule with GMS staining	TMP-SMX	Improved
Saunier et al. 2022 [2]	Lungs, bacteremia, left adrenal gland abscess, brain abscesses	Failure to thrive, severe respiratory distress, hypoxia	77-year-old male, no past medical history, construction worker	Cyriacigeorgica	Blood cultures, MALDI-TOF MS	Linezolid, cefotaxime, amikacin -> cefotaxime, TMP-SMX -> cefotaxime, clarithromycin -> doxycycline, clarithromycin	Improved
Pender et al. 2022 [3]	Lungs, left adrenal gland abscess	Left upper quadrant abdominal pain, fever, diaphoresis, weight loss, nausea and vomiting	57-year-old male, hypertension, poorly controlled diabetes, former smoker	Beijingensis	Tissue culture from left adrenalectomy, 16s rRNA sequencing	TMP-SMX, linezolid -> TMP-SMX; left adrenalectomy	Improved
Langmaid et al. 2021 [1]	Lungs, left adrenal gland abscess	Fever, cough, left-sided abdominal pain	35-year-old male, farmer	Cyriacigeorgica	Blood culture, tissue culture from left adrenal mass, retroperitoneal culture, BAL culture	TMP-SMX, meropenem -> TMP-SMX, ceftriaxone -> TMP-SMX; left adrenalectomy	Improved
Jackson et al. 2018 [10]	Bilateral adrenal gland abscesses, bacteremia, tricuspid valve endocarditis	Fever, abdominal pain, weakness, orthostasis	39-year-old male, splenectomy from splenic laceration, IVDU, HCV	Farcinica	Blood cultures, fluid cultures from percutaneous drainage of adrenal abscesses	TMP-SMX, meropenem -> TMP-SMX; bilateral percutaneous drainage of adrenal abscesses	Improved but lost to follow-up upon discharge
Jackson et al. 2017 [4]	Left adrenal gland abscess, lungs, brain abscesses, bacteremia, urine, bone marrow	Fever, left upper quadrant abdominal pain, weight loss, diarrhea	69-year-old male, coronary artery disease, smoker, prison volunteer	Farcinica	Blood culture, urine culture and bone marrow culture, adrenal gland biopsy	TMP-SMX, meropenem -> TMP-SMX	Improved
de Montmollin et al. 2012 [6]	Right adrenal gland abscess, bacteremia	Fever, right lumbar back pain	59-year-old female, chronic HCV, ileal lymphoma (treated with surgery and radiation), post-radiation enteritis with extensive small bowel resection, ESRD on dialysis, TPN dependent	Farcinica	Fluid culture from aspiration of adrenal abscess, blood cultures, 16 rRNA sequencing, RNA polymerase beta subunit sequencing	Amikacin, cefuroxime -> cotrimoxazole; partial abscess drainage	Improved with treatment but passed from unrelated vascular complication
Al-Tawfiq et al. 2010 [11]	Brain abscesses, bacteremia, lymph nodes, bilateral adrenal gland abscesses	Fever, left upper quadrant abdominal pain	66-year-old female, type 2 diabetes, hypertension, psoriasis, and recently on alefacept and infliximab	Farcinica	Blood culture, fluid culture from aspiration of left adrenal lesion, DNA sequencing	TMP-SMX, linezolid	Deceased
Tachezy et al. 2009 [7]	Lungs, right adrenal gland abscess, brain abscesses	Fever, cough, weakness, nausea	71-year-old female, hypertension, heart failure, former alcoholic	Farcinica	BAL culture, 16s rRNA sequencing	Imipenem, cilastatin, amikacin ->(+ TMP-SMX) -> TMP-SMX	Improved
Chong et al. 2004 [12]	Left adrenal gland abscess	Fever, left loin pain	34-year-old male, new HIV diagnosis with CD4 count of 12 cells/µL	Asteroides	Fluid culture from laparoscopic drainage of abscess	Ceftriaxone, cotrimoxazole -> cotrimoxazole	Improved

Adrenal abscesses should be strongly considered in patients with adrenal masses and ambiguous clinical findings. Given the rarity of nocardial adrenal gland abscesses, maintaining a high index of suspicion in the appropriate clinical context is crucial, as *Nocardia* can present with non-specific symptoms and no identifiable risk factors, as shown in our case. This is particularly important for immunocompromised patients, those with elevated inflammatory markers and rapidly progressing imaging findings despite broad-spectrum antibiotics, and/or concurrent ring-enhancing brain lesions on neuroimaging. In such scenarios, it is advisable to obtain blood cultures, sample potential infection sites for culture, and promptly initiate empiric antimicrobial therapy with coverage for atypical organisms, including *Nocardia*. If clinical suspicion for *Nocardia* remains high despite initial negative results, consulting your hospital's laboratory for further guidance on available testing options is recommended, as advancements in testing methods such as polymerase chain reaction testing, metagenomic next-generation sequencing, and matrix-assisted laser desorption/ionization time-of-flight mass spectrometry have shown increased accuracy and timeliness in detecting *Nocardia* [5,13].

In our case, empiric antimicrobial therapy for disseminated nocardiosis with CNS involvement would have commenced with intravenous administration of trimethoprim-sulfamethoxazole, imipenem, and amikacin for a duration of at least six to nine weeks, with the transition to oral antibiotics contingent upon susceptibilities and clinical and radiographic improvement [15,17]. The use of multiple agents upfront is warranted due to the diverse nature of *Nocardia* species and their varying patterns of antimicrobial susceptibility [15,17,18]. The anticipated duration of treatment would have been a minimum of one year, though ultimately determined by the patient's response to therapy [17,18]. In cases where abscesses do not respond to antimicrobial therapy, surgical intervention and/or percutaneous drainage should be considered if feasible [17].

## Conclusions

We present a rare case of disseminated *Nocardia cyriacigeorgica* that mimicked a metastatic adrenal gland malignancy in an otherwise healthy patient. To our knowledge, this is only the third reported case of *Nocardia cyriacigeorgica* involving the adrenal gland. Given the* *tendency of *Nocardia *to present atypically with non-specific symptoms and abscesses that resemble masses, maintaining a high index of suspicion is crucial for accurate diagnosis and timely management. Nocardiosis affecting the adrenal gland is extremely rare, with only 11 documented cases in the literature. However, it must be considered a diagnostic possibility in the appropriate clinical context, such as in immunocompromised patients, those with concurrent ring-enhancing brain lesions, or the rapid progression of masses despite antimicrobial therapy. Due to its high morbidity and mortality, if clinical findings are equivocal, it is essential to promptly obtain samples from potential infectious sites for culture and molecular studies, as negative blood cultures alone are not always reliable in disseminated nocardiosis.
